# Are Distressed Black Women Also Depressed? Implications for a Mental Health Paradox

**DOI:** 10.1007/s40615-022-01313-7

**Published:** 2022-05-12

**Authors:** Millicent N. Robinson, Christy L. Erving, Courtney S. Thomas Tobin

**Affiliations:** 1 Department of Community Health Sciences (Office 21-245), Jonathan and Karin Fielding School of Public Health, University of California, Los Angeles (UCLA), 650 Charles E. Young Dr. South, Los Angeles, CA 90095 USA; 2 Department of Sociology, College of Arts and Science, Vanderbilt University, Nashville, TN USA

**Keywords:** Black women, Mental health paradox, Depressive symptoms, Major depressive disorder, Psychological distress

## Abstract

**Purpose:**

Recent research suggests the determinants of and links between psychological distress and psychiatric disorder are distinct among Black Americans. Yet, these associations have not been explored among Black women, despite the unique social experiences, risks, and mental health patterns they face. The present study assessed the sociodemographic and psychosocial determinants of distress and disorder and evaluated the distress–disorder association, including whether it was conditional on sociodemographic and psychosocial characteristics among Black women.

**Methods:**

Data were from 328 Black women in the Nashville Stress and Health Study, a cross-sectional community epidemiologic survey of Blacks and Whites in Nashville, Tennessee, and was used to assess the correlates of distress (CES-D depressive symptoms scale) and major depressive disorder (MDD; based on the CIDI). Multinomial logistic regression models estimated the extent to which greater distress was associated with higher risk of “chronic” or “resolved MDD”.

**Results:**

Stress exposure and marital status were associated with greater distress, while stress exposure and childhood SES were associated with elevated disorder risk. Although increased distress was associated with greater disorder risk, significant interactions indicated these associations depend on differences in age and adult socioeconomic status within this population.

**Conclusions:**

This study identifies distinct correlates of distress and disorder and shows that the distress–disorder association varies among subgroups of Black women. Results have important implications for public health research and practice, as they highlight the factors that matter most for the mental health outcomes of Black women.

**Supplementary Information:**

The online version contains supplementary material available at 10.1007/s40615-022-01313-7.

## Introduction

Evidence from epidemiological studies demonstrate paradoxical health patterns among Black Americans. For example, Black Americans generally report lower rates of psychiatric disorder (e.g., major depressive disorder, MDD), but similar or higher levels of psychological distress (e.g., depressive symptoms) compared to White individuals [1–5]. These patterns are counterintuitive because scholars have traditionally thought that psychological distress and psychiatric disorders have shared etiologies [2, 6] and that distress is a risk factor for the onset of major depression [2, 7]. Moreover, Black Americans face greater lifetime exposure to sociodemographic and psychosocial risk factors (i.e., financial strain, racism) [8, 9], which have been linked to elevated rates of both major depression and depressive symptoms in prior research [4, 10, 11]. Thus, these inconsistencies suggest that distress and disorder may arise through independent causal mechanisms for Black Americans and that psychological distress does not consistently predict psychiatric disorders among this population [12]. Indeed, recent scholarship has demonstrated that these outcomes have distinct etiologies [7] that vary between and within social groups [12, 13]. In addition, a recent study found that although distress is associated with increased risk of disorder among White Americans, the extent to which distress contributes to greater odds of chronic major depression depends on the level of social stress exposure across subgroups of Black Americans [12]. These patterns collectively underscore differences in the significance of these factors for Black Americans. Consequently, there is a need to clarify the factors shaping distress and disorder among Black Americans and the association between distress and disorder among this population.

The determinants of distress and disorder and the linkages between distress and disorder have not been widely examined among subgroups of Black Americans, particularly Black women. This gap in the literature is glaring, as growing evidence demonstrates that the “race paradox in mental health” (i.e., the mental health advantage observed among Black vis-à-vis White Americans) persists among women [13–15]. Moreover, although Black women face distinct risks that may contribute to the unexpected health patterns characteristic of the mental health paradox [16], there has been limited focus on understanding mechanisms driving incongruent patterns between psychological distress and psychiatric disorder among Black women. Given that much of what has been demonstrated regarding the links between distress and disorder has been guided by a Eurocentric lens, it is important to explore and clarify these connections among Black women to gain a more comprehensive understanding of this phenomenon and the factors that may shape it, which can ultimately inform efforts to promote well-being among this group.

Social Stress Theory (SST), a theoretical framework used to clarify population differences in mental health, posits that Black Americans will have poorer mental health outcomes compared to White counterparts, given their exposure to low SES, physical health challenges, and increased exposure to social stress [17–20]. Increasing recognition of varied etiologies of distress and disorder, along with the distinct risk factors faced by Black women, underscores the need to clarify the extent to which these factors may differentially shape distress and disorder among this population. Due to their dual race and gender positionality at the intersections of racism and sexism [17, 21–23], Black women face gendered racism, which directly and indirectly impacts the social allocation of resources, along “racially and ethnically ascribed understandings of masculinity and femininity as well as along gendered forms of race and ethnic discrimination” [24]. This social positioning may also contribute to distinct risks, resources, and mental health outcomes among Black women, who face unique challenges as they navigate multiple systemic oppressions [13, 25–28]. For instance, U.S. Black women face disproportionate rates of poverty. Despite comprising only 13 percent of U.S. women, they are reportedly 22 percent of women living in poverty [29]. Encountering consistent financial strain is a chronic stressor, and this stress ultimately shapes poor mental health [4]. Relatedly, a recent study showed that the association between SES and mental health differs by gender for Black Americans, with some evidence suggesting that SES (specifically income) may be more mental health protective for Black women [30].

Black women also experience high levels of social stress exposure across the life course [28, 31, 32], and studies suggest these exposures contribute to poor mental health outcomes (i.e., depressive symptoms and major depression) among this population [13, 31, 33]. In addition to these sociodemographic and psychosocial factors, other characteristics, such as age and marital status [12, 34, 35], may be particularly influential for Black women’s mental health. Despite facing distinct challenges (i.e., financial strain and lower social support), which may contribute to increased risk of major psychological difficulties as they grow older [14, 36], Black women generally experience healthy psychological well-being in older adulthood. One exception is that Black women report elevated depressive symptoms in older adulthood [37]. Findings for the relationship between marital status and mental health among Black women are mixed, such that some indicate that marriage provides psychological benefits for Black women, such as lower risk for depressive symptoms [25, 38], while others indicate that marital status is not associated with mental health among Black women [39]. Notwithstanding, there has been limited consideration allocated to clarifying how the aforementioned factors may differentially contribute to risk of psychological distress relative to psychiatric disorders or how they may jointly shape the association between distress and disorder among Black women.

Although non-specific psychological distress is thought to be associated with the onset of psychiatric disorder among the general population [2, 6], prior research suggests this association may also vary significantly among Black Americans [7, 12, 40]. Considering the past and present sociopolitical context shaping the lived experiences of Black Americans in the United States, particularly in terms of navigating various forms of systemic oppression, it would stand to reason that this social landscape may uniquely inform Black Americans’ perceptions of distress and subsequent disorder. Despite a lower risk of psychiatric disorder, even when Black Americans are diagnosed with psychiatric disorders, they often report more severe and debilitating symptoms compared to White Americans [12, 31, 41]. Nevertheless, this has not been assessed among Black women specifically. This begs the question, *“Are distress and disorder associated among Black women?”*. Given the unique social experiences, risks, and mental health patterns of Black women specifically, the links between distress and disorder may be especially distinct among this group. For instance, prior research has demonstrated that racialized-gendered stressors are associated with psychological distress among Black women [33]. Additionally, due to factors such as experiences of gendered racism, lack of culturally sensitive providers, and societal stigma, Black women report a lower utilization of mental healthcare services, despite reporting high levels of psychological distress [32]. Given these issues, Black women are often undiagnosed or misdiagnosed because they are societally perceived as “Strong Black Women” and are not afforded the care they need. Collectively, these circumstances likely facilitate conditions that increase this group’s risk for more severe diagnoses and symptoms [32]. Furthermore, as recent research challenges the assumption of similarity and demonstrates that the effects of sociodemographic and psychosocial factors vary by race and gender [42, 43], it is possible that these factors may also exacerbate the effects of distress on disorder among Black women. However, this hypothesis has not been empirically evaluated.

To this end, the purpose of the present study is to explore the distinctions between distress and disorder among Black American women. More specifically, the aims are to: (1) assess the sociodemographic and psychosocial determinants of distress and disorder; (2) examine the relationship between distress and disorder; and (3) evaluate whether the distress–disorder association is conditional on sociodemographic and psychosocial characteristics among Black women. Clarifying these patterns will assist in determining specific pathways for intervention to promote psychological well-being.

## Methods

The Nashville Stress and Health Study (NSAHS) is a population-based sample of Black and White adults, ages 21 to 69, from the city of Nashville and surrounding areas within Davidson County, Tennessee. A random sample was obtained using a multistage, stratified sampling approach. Black households were oversampled to achieve a final sample with similar proportions of racial and sex groups, and a sampling weight allowed for generalizability of sample characteristics to the county population. American Association for Public Opinion Research (AAPOR) rates were used to evaluate success across screening and interviewing phases (Response Rate 1 = 30.2). Between 2011 and 2014, 1,252 respondents were interviewed about their personal and family backgrounds, stress and coping experiences, health behaviors, and health histories during three-hour computer-assisted interviews with trained study staff of the same race. All participants provided informed consent. Study procedures were approved by the Vanderbilt University Institutional Review Board and have been described in detail elsewhere [44]. The original sample size included 330 Black women. However, two cases were missing key study variables. Thus, the present analyses include data from 328 Black women and sample characteristics are provided in Table 1.

**Table 1:** Sample Characteristics among Black Women, Nashville Stress and Health Study (2011–2014; N = 328)

	*Mean or %*	*(SD)*	*[Range]*
Health Outcomes			
Depressive Symptoms	16.02	(12.92)	[0–47]
Major Depressive Disorder (MDD)			
No MDD (Ref.)	74.72		
Resolved MDD	13.21		
Chronic MDD	12.08		
Sociodemographic & Psychosocial Factors			
Age	43.69	(14.71)	[26-69]
Marital Status			
Married (Ref.)	26.33		
Never Married	44.31		
Other	29.36		
Childhood Socioeconomic Status (CSES)^1^	-0.42	(1.01)	[-2.72–1.78]
Adult Socioeconomic Status (ASES)^1^	-0.46	(0.98)	[-2.93–1.68]
Stress Exposure^1^	0.78	(3.73)	[-4.92–13.05]

Weighted means and percentages are presented. SD = standard deviation; Ref. = reference category; ^1^Standardized variable

### Dependent Measures

*Depressive symptoms* were measured using the 20-item (Cronbach’s alpha = 0.89) version of the Center for Epidemiologic Studies-Depression (CES-D) scale that assessed past-month depressive symptoms [45, 46]. Examples of items include, “could not shake off the blues,” “felt depressed,” “sleep was restless,” and “had crying spells.” Response categories included (0) not at all, (1) occasionally, (2), frequently, and (3) almost all the time. Items were summed such that scores ranged from 0 to 60, with higher values indicating higher depressive symptoms.

*Major depressive disorder (MDD)* was evaluated with the World Mental Health Survey Initiative version of the World Mental Health Composite International Diagnostic Interview (WMH-CIDI), which assesses the most common and severe mental disorders using diagnostic criteria established by the Diagnostic and Statistical Manual of Mental Disorders, Fourth Edition [47, 48]. Lifetime and past-year prevalence of MDD were assessed, and a categorical variable was created to account for the chronicity of MDD [12, 41]: (0) No MDD (no lifetime or past-year MDD; reference category); (1) Resolved MDD (lifetime, but no past-year MDD); (2) Chronic MDD (lifetime and past-year MDD).

### Independent Measures

#### Sociodemographic and Psychosocial Factors

*Childhood socioeconomic status (CSES)* was a standardized index of three dimensions: parental educational attainment (e.g., less than high school, high school/ general education degree (GED), some college, and college graduate or higher), family’s financial situation most of the time while growing up (e.g., “often struggled to pay for food, clothing, and shelter”), and parental occupational prestige (range = 0–100; based on the Nam-Powers-Boyd occupational status scale [49]. CSES scores were calculated by first standardizing and summing each dimension and then dividing the total by the number of available dimensions to avoid potential data loss [50]. This resulted in continuous CSES scores with the mean score set to zero; values greater than zero corresponded with “above average” CSES and values below zero indicated “below average” CSES levels. *Adult Socioeconomic Status (ASES)* scores were generated using a similar approach. For each respondent, (1) level of educational attainment, (2) annual household income, and (3) occupational prestige score were standardized, summed, and divided by the number of available dimensions. ASES was measured continuously, such that scores above zero corresponded with above average ASES.

*Stress exposure* was assessed with five dimensions of social stress: recent life events, chronic stress, trauma, everyday discrimination, and major discrimination. Recent life events were measured using a count of 32 stressful events (e.g., “Did a child die?” “Was there a marital separation or divorce?”) experienced by respondents or someone close to them within the past 12 months. Chronic stress was based on 36 items across several domains such as employment, relationships, and general strain. Respondents were asked the extent to which each item is true (0 = not true; 1 = somewhat true; 2 = very true); items were summed such that higher values corresponded with higher levels of chronic stress. Lifetime trauma was measured using 43 major and potentially traumatic events (0 = no, 1 = yes), including childhood maltreatment, witnessed and experienced violence, and sexual abuse [51]. Major discrimination [4] was assessed with a count of seven major events of unfair treatment (e.g., “been unfairly treated by the police”) across the life course (0 = no, 1 = yes). Everyday discrimination was measured with the nine-item (Cronbach's alpha = 0.86) “Everyday Discrimination Scale” [4], which assesses the frequency of “everyday” slights and hassles such as, “you receive worse service than other people at restaurants or stores” (0 = never to 4 = almost always). Items were summed such that higher values indicated more chronic experiences with discrimination. To measure total stress exposure, each dimension was standardized and summed so that zero represented the mean; scores less than zero indicated below average exposure and scores above zero indicated higher than average exposure. Past research has also utilized composite stress measures to capture the influence of stress overall on health [52, 53].

Respondents’ *age* (ranged from 22 to 69 years) and current *marital status* (0 = married, 1 = never married, 2 = other [i.e., separated, widowed, divorced; “formerly married”]) were also examined.

#### Analysis Plan

**Fig. 1:**
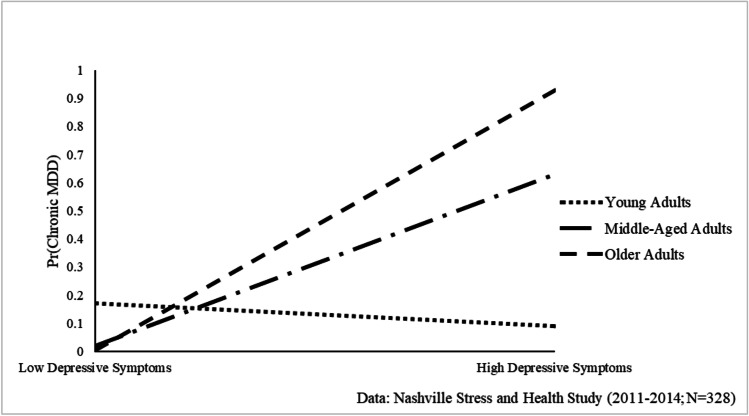
Age Moderates the Relationship between Depressive Symptoms and Chronic Major Depressive Disorder (MDD) among Black Women

**Fig. 2:**
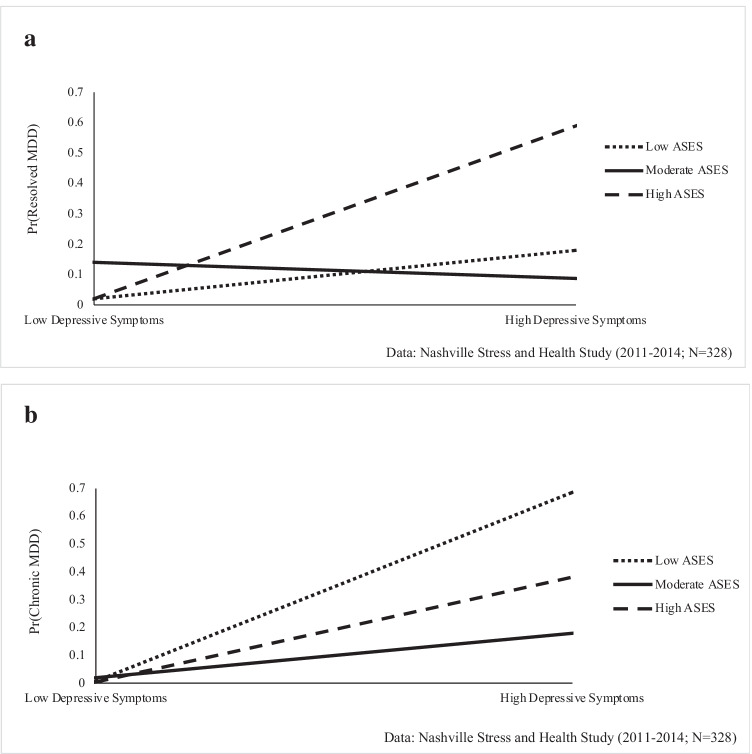
**A** Adult Socioeconomic Status (ASES) Categories Moderate the Relationship between Depressive Symptoms and Resolved Major Depressive Disorder (MDD) among Black Women, **B** Adult Socioeconomic Status (ASES) Categories Moderate the Relationship between Depressive Symptoms and Chronic Major Depressive Disorder (MDD) among Black Women

First, weighted means and proportions were estimated for key dependent and independent variables. Second, the sociodemographic and psychosocial correlates associated with depressive symptoms and MDD were assessed. Ordinary least squares (OLS) regression models estimated relationships with depressive symptoms, and unstandardized regression coefficients and standard errors are presented. Multinomial logistic regression estimated the odds of “resolved MDD” and “chronic MDD” relative to “no MDD.” Third, the association between depressive symptoms and MDD was examined, accounting for sociodemographic and psychosocial factors. Last, interactions between depressive symptoms and each sociodemographic and psychosocial factor were tested to determine the conditional impact of each factor on the depressive symptoms-MDD association. Significant interactions are presented in Figs. 1–2. Analyses were conducted using STATA 15.1.

## Results

### Descriptive Statistics

Sample characteristics are presented in Table 1. Black women had an average depressive symptom score of 16.02 (SD = 12.92), slightly above the clinical significance threshold of 16 [44]. While most women reported no MDD (74.72%), 13.21% had resolved MDD and 12.08% had chronic MDD. CSES (m = -0.42, SD = 1.01) and ASES (m = -0.46, SD = 0.98) varied among women, with most reporting low-moderate socioeconomic resources in childhood and adulthood. Similarly, stress exposure varied among women, with most reporting moderate to high levels of social stressors (m = 0.78, SD = 3.73). The average age was 43.69 years (SD = 14.71). While 44.31% had never married, 26.33% were married and 29.36% were formerly married (i.e., separated, widowed, divorced).

### Determinants of Depressive Symptoms and MDD

The sociodemographic and psychosocial correlates of depressive symptoms and MDD among Black women are assessed in Table 2. Increased exposure to social stressors was linked to significantly higher depressive symptoms (b = 1.87, SE = 0.26, p < 0.001). Relative to married women, never married (b = 4.04, SE = 1.12, p < 0.001) and formerly married women (b = 4.40, SE = 1.49, p < 0.01) also experienced elevated depressive symptoms. Age, CSES, and ASES were not associated with depressive symptoms. Nevertheless, the sociodemographic and psychosocial factors examined collectively explained 38% of the variation in depressive symptoms.

**Table 2:** Sociodemographic and Psychosocial Determinants of Depressive Symptoms and Major Depressive Disorder (MDD) among Black Women, Nashville Stress and Health Study (2011–2014; N = 328)

	Depressive Symptoms			Major Depressive Disorder MDD (Ref. = No MDD)					
				Resolved MDD^a^			Chronic MDD^a^		
	*b*	*(SE)*	*p-Value*	*RRR*	*(95% CI)*	*p-Value*	*RRR*	*(95% CI)*	*p-Value*
Sociodemographic & Psychosocial Factors									
Age	-0.11	(0.06)	p = 0.06	1.02	(0.96, 1.09)	p = 0.50	1.02	(0.96, 1.08)	p = 0.58
Marital Status (Married = 1)									
Never Married	4.04	(1.12)	p < 0.001	3.01	(0.72, 12.54)	p = 0.13	0.95	(0.13, 7.15)	p = 0.96
Other	4.40	(1.49)	p < 0.01	2.62	(0.47, 14.75)	p = 0.27	0.82	(0.08, 7.84)	p = 0.86
Childhood Socioeconomic Status (CSES)	0.50	(1.49)	p = 0.60	1.17	(0.52, 2.65)	p = 0.70	2.36	(1.30, 4.28)	p < 0.01
Adult Socioeconomic Status (ASES)	-1.65	(1.31)	p = 0.21	1.34	(0.80, 2.26)	p = 0.26	0.47	(0.22, 1.01)	p = 0.05
Stress Exposure	1.87	(0.26)	p < 0.001	1.34	(1.14, 1.56)	p < 0.001	1.55	(1.33, 1.81)	p < 0.001
Intercept	15.53	(2.41)	p < 0.001	0.03	(0.002, 1.56)	p < 0.001	0.04	(0.002, 0.91)	p < 0.05
	R^2^ = 0.38			F = 7.23, p < 0.001					

For depressive symptoms, unstandardized regression coefficients *(b)* and standard errors *(SE)* are presented. Relative risk ratios (RRR) and 95% confidence intervals (CI) are presented for major depressive disorder (MDD). ^a^"No MDD" is the referent group.

MDD was influenced by different factors. Specifically, only stress exposure was linked to resolved MDD: every one-point increase in stress exposure scores contributed to 34% greater risk of resolved MDD relative to no MDD. CSES (RRR = 2.36, 95% CI = 1.30–4.28, p < 0.01) and stress exposure (RRR = 1.55, 95% CI = 1.33–1.81, p < 0.001) were associated with significantly greater risk of chronic MDD relative to no MDD.

### Associations between Depressive Symptoms and MDD

The relationship between depressive symptoms and MDD is examined in Table 3. Results indicate that depressive symptoms were significantly associated with increased risk resolved MDD (RRR = 1.07, 95% CI = 1.03–1.12, p < 0.001) and chronic MDD (RRR = 1.13, 95% CI = 1.07–1.20, p < 0.001). Table 4 shows that this association was conditional on age (RRR = 1.01, 95% CI = 1.01–1.02, p < 0.05). As illustrated in Fig. 1, the depressive symptoms–MDD association amplifies with age, such that depressive symptoms were linked to minimal risk of chronic MDD among young women. In contrast, increases in depressive symptoms were associated with heightened risk of chronic MDD for middle-aged and older women.

**Table 3:** The Relationship between Depressive Symptoms and Major Depressive Disorder (MDD) among Black Women, Nashville Stress and Health Study (2011–2014; N = 328)

	Major Depressive Disorder MDD (Ref. = No MDD)					
	Resolved MDD^a^			Chronic MDD^a^		
	*RRR*	*(95% CI)*	*p-Value*	*RRR*	*(95% CI)*	*p-Value*
Depressive Symptoms	1.07	(1.03, 1.12)	p < 0.001	1.13	(1.07, 1.20)	p < 0.001
Age	1.03	(0.96, 1.10)	p = 0.37	1.03	(0.96, 1.12)	p = 0.39
Marital Status (Married = 1)						
Never Married	2.21	(0.55, 8.87)	p = 0.26	0.48	(0.05, 4.65)	p = 0.52
Other	1.79	(0.33, 9.74)	p = 0.50	0.40	(0.05, 3.44)	p = 0.40
Childhood Socioeconomic Status (CSES)	1.20	(0.51, 2.78)	p = 0.68	2.20	(1.16, 4.17)	p < 0.01
Socioeconomic Status (ASES)	1.47	(0.96, 2.25)	p = 0.08	0.59	(1.16, 1.49)	p = 0.27
Stress Exposure	1.47	(0.13, 1.40)	p < 0.05	1.31	(1.07, 1.61)	p < 0.01
	F = 17.09, p < 0.001					

Relative risk ratios (RRR) and 95% confidence intervals (CI) are presented.

**Table 4:** The Conditional Impact of Psychosocial/Sociodemographic Fators on the  Depressive Symptoms- Major Depressive Disorder (MDD) Association among Black Women, Nashville Stress and Health Study (2011-2014; N=328)

	Major Depressive Disorder MDD (Ref.=No MDD)					
	***Resolved MDD***^***a***^			***Chronic MDD***^***a***^		
	*RRR*	*(95% CI)*	*p-Value*	*RRR*	*(95% CI)*	*p-Value*
**Model 1**						
Age	0.99	(0.90, 1.10)	p=0.91	0.90	(0.76, 1.06)	p=0.19
Depressive Symptoms	0.96	(0.85, 1.10)	p=0.59	0.86	(0.67, 1.10)	p=0.23
Depressive Symptoms x Age	1.00	(0.99, 1.01)	p=0.14	1.01	(1.01, 1.02)	p<0.05
**Model 2**						
Marital Status (Married=1)						
Never Married	0.55	(0.10, 3.12)	p=0.50	7.94	(0.18, 349.49)	p=0.28
Other	0.48	(0.04, 6.41)	p=0.58	0.06	(0.001, 4.36)	p=0.20
Depressive Symptoms	0.99	(0.87, 1.13)	p=0.88	1.23	(1.02, 1.50)	p<0.05
Marital Status x Depressive Symptoms						
Depressive Symptoms x Never Married	1.10	(0.96, 1.25)	p=0.17	0.88	(0.70, 1.11)	p=0.28
Depressive Symptoms x Other	1.10	(0.94, 1.29)	p=0.21	1.05	(0.83, 1.33)	p=0.68
**Model 3**						
Childhood Socioeconomic Status (CSES)	2.84	(0.71, 11.30)	p=0.14	3.77	(1.02, 13.95)	p<0.05
Depressive Symptoms	1.06	(1.02, 1.11)	p<0.01	1.13	(1.06, 1.20)	p<0.001
Depressive Symptoms x CSES	0.96	(0.90, 1.02)	p=0.17	0.97	(0.92, 1.03)	p=0.39
**Model 4**						
Adult Socioeconomic Status (ASES)	1.63	(0.83, 3.20)	p=0.16	0.87	(0.22, 3.34)	p=0.84
Depressive Symptoms	1.07	(1.03, 1.12)	p<0.001	1.12	(1.05, 1.20)	p<0.001
Depressive Symptoms x ASES	0.99	(0.96, 1.03)	p=0.76	0.98	(0.94, 1.02)	p=0.41
**Model 5**						
Stress Exposure	1.22	(0.97, 1.53)	p=0.09	1.84	(1.17, 2.92)	p<0.01
Depressive Symptoms	1.07	(1.02, 1.13)	p<0.01	1.17	(1.09, 1.26)	p<0.001
Depressive Symptoms x Stress Exposure	1.00	(0.99, 1.01)	p=0.80	0.99	(0.97, 1.00)	p=0.10

Notes: Relative risk ratios (RRR) and 95% confidence intervals (CI) are presented. All models adjust for age, marital status, CSES, ASES, and stress exposure.

While the interaction between depressive symptoms and continuous ASES was non-significant (see Table 4, Model 4), supplemental analyses indicated the conditional impact of ASES may be subject to threshold effects. Specifically, when examining the interaction between depressive symptoms and ASES measured categorically (i.e., low, moderate, high ASES), results show that the depressive symptoms-resolved MDD association was diminished among women with moderate ASES relative to those with low ASES (RRR = 0.89, 95% CI = 0.81–0.98, p < 0.01). This pattern is depicted in Fig. 2A, which shows that increases in depressive symptoms were linked to significantly higher risk of resolved MDD for high ASES women relative to low ASES women. For those with moderate ASES, there was not a significant relationship between depressive symptoms and resolved MDD. The relationship between depressive symptoms and chronic MDD was similarly diminished among those with moderate ASES relative to high ASES women (RRR = 0.89, 95% CI = 0.80–0, 0.99, p < 0.05). However, as shown in Fig. 2B, the link between depressive symptoms and chronic MDD was amplified among women with low ASES and high ASES, but non-significant among moderate ASES women.

## Discussion

Notwithstanding evidence from epidemiological studies demonstrating paradoxical health patterns among Black Americans, the relationship between distress and disorder and the factors that may shape this relationship among Black women have not been previously examined. Using a community sample of Black women living in the Nashville area, this study had three aims. First, to describe the sociodemographic and psychosocial determinants of depressive symptoms and MDD. Second, to establish whether there was a relationship between depressive symptoms and MDD. Third, to assess whether the distress–MDD association was conditional on sociodemographic and psychosocial factors. Several key findings are described below. We then offer insights for future research and intervention strategies for reducing mental health challenges among Black women.

First, results revealed both similar and unique determinants of distress and MDD among Black women. Stress exposure was associated with higher depressive symptoms and increased odds for resolved and chronic MDD. As Black women navigate gendered racism [23, 54], stress exposure is often unavoidable and chronic [55, 56]. In turn, ongoing stress contributes to the development of both psychological distress and more severe mental health challenges. However, marital status was a unique sociodemographic correlate of depressive symptoms: that is, married Black women had fewer depressive symptoms relative to their unmarried counterparts. Other research has also identified the health protectiveness of marriage for Black women despite their lower marriage rates in comparison with White and other ethnic minority women [57]. The broader implication is that among unmarried women, other sources of social support should be identified to reduce depressive symptoms. Though unanticipated, a significant social correlate of chronic MDD was high childhood SES. This finding is a sharp departure from research reporting high childhood SES as a protective mechanism for mental health [58], but it is consistent with research showing a positive association between high SES and depression among Black men and adolescents [59, 60]. Diminished health returns of high childhood SES among Black Americans have also been observed for other health domains, including body mass index [61]. Black children from higher socioeconomic backgrounds may be exposed to majority-White environments where they experience racial discrimination at higher rates with less social support and other psychosocial resources to address these challenges. The stress and strains of middle-class status among Black professionals reveal similar challenges with tokenism and devaluation in White workplaces [62]. Such environments are psychologically taxing and socially isolating. Similar dynamics may also be operant for Black women raised in middle and upper middle-class families.

Second, distress was associated with MDD among Black women. Though non-specific psychological distress is thought to be associated with the onset of psychiatric disorder among the general population, prior research suggests this association could vary significantly among Black Americans [2]. As such, confirming this association among Black women is a contribution to the broader literature, as within-group differences among Black Americans are rarely topics of investigation in this research area. In addition, our study results are aligned with a recent study that reported a significant association between distress and disorder among Black Americans [12].

Third, age moderated the association between distress and chronic MDD such that the influence of distress on chronic MDD was weak to non-existent among young adults (18–35), but strong and positive among midlife (36–49) and later life adults (50 +). Black American women experience distinct inequalities as they age, which taken together may contribute to this population being more vulnerable to chronic MDD in later life. As a result of the accumulation of compounded stress experienced over the entire life course [63], older Black women are the most likely to be living alone (43%) [64] have the lowest levels of wealth accumulation [36], highest rates of widowhood [36], and the most precarious retirement benefits [65] when compared to Black men and White individuals. Hence, age and aging processes among Black women may influence the extent to which depressive symptoms will induce chronic mental health challenges later in the life course.

Fourth, adult socioeconomic status moderated the distress–MDD association. Consistent with the diminished returns hypothesis suggesting that Black Americans accrue limited health benefits from higher SES [59, 66–68], the association between distress and *resolved* MDD was strongest for Black women of high adult socioeconomic status. This suggests that for high-SES women, high distress levels increase their susceptibility to past MDD, whereas this pattern is not observed for women with currently moderate or low SES. Black individuals occupying elite occupational positions are subject to social support loss [43], tokenism [69], and potential detachment from their racial identity. For instance, Black women in majority-White professional settings often must engage in “shifting,” changing their appearance and speech to appear acceptable or favorable to White individuals' sensibilities [70, 71]. This version of “double consciousness” could act as a catalyst for distress to develop into disorder among high-status Black women. Nevertheless, their high SES status may enable them to mobilize resources (e.g., psychological counseling services) to eventually resolve MDD. On the other hand, the association between distress and *chronic* MDD was strongest for women of low adult socioeconomic status. This finding suggests that for currently low-SES women, depressive symptoms are linked to more chronic, and ongoing symptoms. With access to fewer economic resources that would enable them to resolve MDD, this finding suggests that the strains of economic vulnerability prevent low-SES Black women from seeking and utilizing mental health services. Moreover, financial strains and material hardship may make it difficult for low-SES Black women to recover from psychological distress which, in turn, contributes to the chronicity of MDD.

Last, one finding departs from previous research on the distress–disorder association: Stress exposure was not a significant moderator [12]. While stress in combination with distress may induce disorder under some circumstances, our findings potentially reveal evidence of resilience among Black women. However, future research should assess the extent to which specific stressors (e.g., financial strain, discrimination) contribute to the association between distress and MDD. Consistent with past research [52, 53], this study focused on a composite measure of stress exposure to ascertain whether stress more generally moderated the relationship between distress and disorder. Nevertheless, despite experiencing high levels of stress exposure and psychological distress, Black women may be able to counteract the pernicious elements of stress through the mobilization of coping resources such as social support [43] and psychological factors such as high self-esteem [43, 48], and maintaining feelings of hope [72]. In sum, distress in tandem with high stress exposure may not develop into disorder because of the creative ways Black women respond to and resist structural inequality.

## Study Limitations

Though the present study provides valuable contributions to the literature, there are a few limitations. This study used cross-sectional data, which makes it difficult to determine causality. Future work exploring the association between distress and disorder among Black women would benefit from longitudinal data and methodologies. Secondly, the data were collected in one county in Tennessee; thus, we are unable to generalize the findings. Future work should explore these questions using a national dataset. Additionally, this study did not assess the impact of gendered racism in shaping distress and disorder among Black women. Given that this form of stressor is ongoing for many Black women, and is an obstacle to well-being among this population [22, 55], future work would benefit from examining the links between gendered racism, distress, and disorder among Black women. Importantly, other intersectional axes of stratification may influence the association between distress and disorder including, but not limited to ethnicity, sexual orientation, and ability status. Last, the present study did not assess the role of coping in shaping distress and disorder. Due to the unrelenting nature of systemically oppressive systems, Black women engage in various forms of coping, such as endorsing the Superwoman Schema or “Strong Black Women” role in efforts to offset these destabilizing forces [31, 32]. Future work would benefit from assessing the role of coping in shaping the distress–disorder link among Black women.

## Implications for Practice and/or Policy

Study findings have implications for intervention development. For example, these results provide more information pertaining to how and at what point to intervene to decrease MDD risk among Black women. Specifically, interventions for resolving or reducing depressive symptoms earlier in the life course are of utmost importance. Given the structural arrangements that operate to the severe disadvantage of older Black women, resolving depressive symptoms earlier in the life course for future generations of Black women could alleviate the strong linkage between distress and disorder when they get older. One possible method of intervention would be providing therapeutic services to Black girls, adolescents, and young adults. However, it is vital that barriers to accessing this care (i.e., stigma, lack of culturally sensitive providers, incorporation of spirituality, cost) are comprehensively addressed and integrated [32]. Implementing such an intervention may help to ensure that Black women are able to successfully navigate mental health challenges across the life course, which could ultimately contribute to their health and well-being.

Though we have focused on MDD in the context of this study, other mental disorders are important to detect and resolve among Black women. Especially because Black women are less likely to meet criteria for MDD (compared to White women) [73], it is essential for primary care providers and other medical professionals to screen for other mental health problems more common among Black women such as post-traumatic stress disorder (PTSD) [13] and dysthymic disorder, a form of low-grade depression that is more chronic than MDD [74]. In sum, the clinical practice implication is that other mental health problems (beyond MDD) should be assessed in the context of Black women’s clinical encounters with health care professionals.

## Conclusion

Considering that prior work has underscored the importance of conducting within-group analysis to elucidate distinct patterns, the present study addresses this gap by shedding new light on the mental health paradox among Black women. We extend prior research by demonstrating that distress and disorder both share and have distinct correlates among Black women. For instance, while some factors (e.g., stress exposure) significantly contribute to both elevated distress levels and disorder risk, others (e.g., marital status and CSES) have distinct influences on distress and disorder among Black women. Interestingly, high SES (childhood and adulthood) does not appear to confer any mental health benefits and may even impose mental health risk for this population. Like prior research, the present study shows that distress is significantly associated with disorder among this group. However, this relationship depends on age and SES, such that distressed Black women who are young and middle-class face limited disorder risk. Prior literature on Social Stress Theory and the mental health paradox has been extended and challenged by these findings, which indicate that the mechanisms operating among the broader population to shape mental health patterns may not operate similarly across or even within groups.

## Supplementary Information

Below is the link to the electronic supplementary material.

Supplementary file1 (DOCX 63 kb)

## Data Availability

Analyses were completed using STATA 15.1 software.

## Data Availability

Data and material are not publicly available.

## Declarations

### Ethnical Approval

All human and animal studies have been approved by the appropriate ethics committee and have therefore been performed in accordance with the ethical standards laid down in the 1964 Declaration of Helsinki and its later amendments. The collection of NSAHS data was approved by the IRB at Vanderbilt University.
