# Supplementary material for: Are Distressed Black Women Also Depressed? Implications for a Mental Health Paradox
Source: J Racial Ethn Health Disparities. 2022 May 12;10(3):1280–92. doi: 10.1007/s40615-022-01313-7 (PMC9652478; doi:10.1007/s40615-022-01313-7)
Supplement: Supplementary file 1 [file 40615_2022_1313_MOESM1_ESM.docx]

Supplemental Table 1. The Conditional Impact of Adult Socioeconomic Status (ASES) Cat[illegible] on the Depressive Symptoms- Major Depressive Disorder (MDD) Association among B[illegible] Women,
Nashville Stress and Health Study (2011-2014; N=328)

	Resolved MDD[a]			Chronic MD[illegible]	
	RRR	(95% CI)	p-Value	RRR	(95% CI)
ASES (Low ASES=1)					
Moderate ASES	8.06	(1.44, 45.21)	p<0.01	5.50	(0.76, 39.83)
High ASES	0.78	(0.06, 9.75)	p=0.85	0.47	(0.02, 8.95)
Depressive Symptoms	1.12	(1.04, 1.18)	p<0.001	1.19	(1.11, 1.27)
Depressive Symptoms x ASES					
Moderate ASES	0.89	(0.81, 0.98)	p<0.01	0.89	(0.80, 0.99)
High ASES	1.07	(0.06, 9.75)	p=0.85	1.04	(0.93, 1.16)
F=3.83, p<0.01					

Notes: Relative risk ratios (RRR) and 95% confidence intervals (CI) are presented. All models adjust for age, marital status, [illegible] stress exposure.
